# Spread of Extensively Drug-Resistant Tuberculosis in KwaZulu-Natal Province, South Africa

**DOI:** 10.1371/journal.pone.0017513

**Published:** 2011-05-31

**Authors:** Prashini Moodley, N. Sarita Shah, Nabihah Tayob, Cathy Connolly, Nicola Zetola, Neel Gandhi, Gerald Friedland, A. Willem Sturm

**Affiliations:** 1 KwaZulu-Natal Research Institute for Tuberculosis and HIV (K-RITH), Durban, South Africa; 2 Department of Infection Prevention and Control, University of KwaZulu-Natal and KwaZulu-Natal Department of Health, Durban, South Africa; 3 Divisions of General Internal Medicine, Infectious Diseases, and Epidemiology, Albert Einstein College of Medicine, Bronx, New York, United States of America; 4 Tugela Ferry Care and Research (TF CaRes) Collaboration, Tugela Ferry, KwaZulu-Natal, South Africa; 5 Medical Research Council, Durban, KwaZulu-Natal, South Africa; 6 Division of Infectious Diseases, University of Pennsylvania, Philadelphia, Pennsylvania, United States of America; 7 Yale AIDS Program, Yale School of Medicine, New Haven, Connecticut, United States of America; Université Pierre et Marie Curie, France

## Abstract

**Background:**

In 2005 a cluster of 53 HIV-infected patients with extensively drug-resistant tuberculosis (XDR-TB) was detected in the Msinga sub-district, the catchment area for the Church of Scotland Hospital (CoSH) in Tugela Ferry, in KwaZulu-Natal province (KZN), South Africa. KZN is divided into 11 healthcare districts. We sought to determine the distribution of XDR TB cases in the province in relation to population density.

**Methods:**

In this cross-sectional study, the KZN tuberculosis laboratory database was analysed. Results of all patients with a sputum culture positive for *Mycobacterium tuberculosis* from January 2006 to June 2007 were included. Drug-susceptibility test results for isoniazid, rifampicin, ethambutol, streptomycin, kanamycin and ofloxacin were available for all patients as well as the location of the hospital where their clinical diagnosis was made.

**Findings:**

In total, 20858 patients attending one of 73 hospitals or their adjacent clinics had cultures positive for *M. tuberculosis*. Of these, 4170 (20%) were MDR-TB cases. Four hundred and forty three (11%) of the MDR tuberculosis cases displayed the XDR tuberculosis susceptibility profile. Only 1429 (34%) of the MDR-TB patients were seen at the provincial referral hospital for treatment. The proportion of XDR-TB amongst culture-confirmed cases was highest in the Msinga sub-district (19.6%), followed by the remaining part of the Umzinyati district (5.9%) and the other 10 districts (1.1%). The number of hospitals with at least one XDR-TB case increased from 18 (25%) to 58 (80%) during the study period.

**Interpretation:**

XDR-TB is present throughout KZN. More than 65% of all diagnosed MDR-TB cases, including XDR-TB patients, were left untreated and likely remained in the community as a source of infection.

## Introduction

Multidrug-resistant (MDR) tuberculosis, including its extensively resistant (XDR) form has become a threat to the success of tuberculosis control programs. MDR tuberculosis is now a global epidemic with over 511000 estimated cases in 2007 [1].

**Figure 1:**
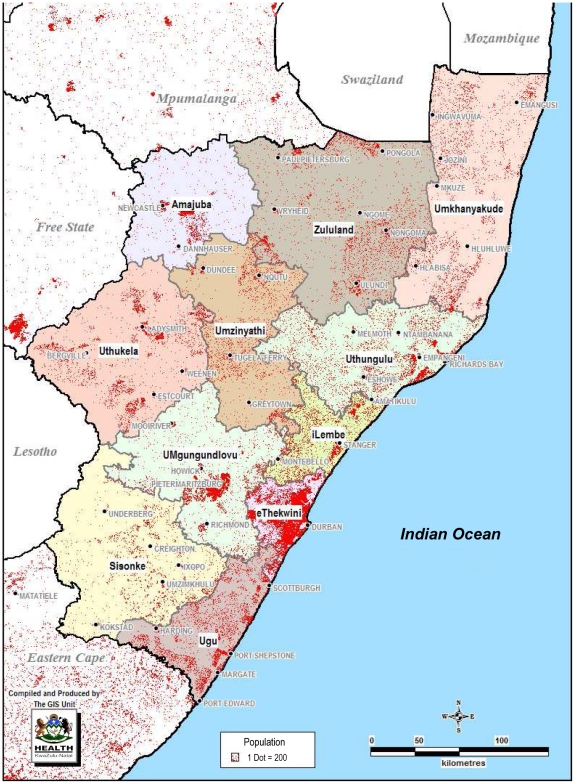
Health districts of KwaZulu-Natal province and population density.

In 2005, 53 cases of extensively drug-resistant (XDR) tuberculosis were detected in the Msinga sub-district, Umzinyati health district, KwaZulu-Natal Province in South Africa (Fig. 1) [2].

All 53 were HIV infected. They sought healthcare at the Church of Scotland Hospital (CoSH) in Tugela Ferry whose catchment area is the Msinga sub-district. This raised the question as to whether this was nosocomial transmission in CoSH, an isolated outbreak in the Msinga community, or a widespread epidemic involving other districts of KwaZulu-Natal Province. The first indication that this was not an isolated nosocomial outbreak in CoSH came from the observation that 30 of the 53 patients were identified through a survey amongst outpatients suspected of having tuberculosis. Fourteen of the 30 had no prior history of hospitalization [2]. While there was evidence of nosocomial and community transmission, the question remained as to how extensive the latter was. Since there was no obvious reason why community spread should be restricted to the Msinga sub-district, we hypothesised that XDR tuberculosis was present throughout KwaZulu-Natal Province.

## Methods

### Study Design

This was a cross-sectional study, examining the culture and drug susceptibility database of the provincial tuberculosis laboratory in KwaZulu-Natal Province.

### Setting

KwaZulu-Natal Province has a population of 9.7 million (2003 census) [3] and is divided in 11 health districts (Fig. 1). According to the provincial register, the incidence of tuberculosis in 2006 was 780 per 100,000 population. The HIV sero-prevalence among antenatal attendees was 38% in 2006 [4].

KwaZulu-Natal Province has 74 public hospitals, with varying numbers of affiliated clinics. Hospitals were grouped according to the district in which they are located. At the time of this study, King George V Hospital (KGV) was the single MDR and XDR tuberculosis referral hospital for patients in KwaZulu-Natal Province. Patients confirmed as being infected with a MDR/XDR strain of *M. tuberculosis* at one of the public hospitals or clinics, were referred to King George V Hospital for further management.

### TB culture and drug-susceptibility testing

The South African national guidelines for diagnosis of tuberculosis allow culture and drug-susceptibility testing to be performed only on specimens from patients with a clinical diagnosis of tuberculosis who failed to respond to first-line therapy, who interrupted treatment, who had a previous diagnosis of tuberculosis or had repeatedly negative smear microscopy results. Adherence to these guidelines depends on accessibility to diagnostic facilities. Of note, after the report of XDR tuberculosis in 2005 [2], providers at CoSH in the Msinga sub-district started to send specimens from all patients suspected of tuberculosis for culture and drug-susceptibility testing.

Drug-susceptibility test results were generated by the 1% proportional method using Middlebrook 7H10 agar plates [5]. In KwaZulu-Natal Province, routine susceptibility testing for *M.tuberculosis* includes the following drugs: isoniazid, rifampicin, ethambutol, streptomycin, kanamycin and ofloxacin.

### Data Source and Data Management

The drug-susceptibility profile of all *M.tuberculosis* isolates from sputum, obtained from patients between 1 January 2006 and 30 June 2007 at the centralised TB laboratory in KwaZulu-Natal Province, were analysed. Specimens from patients attending private hospitals are not sent to this laboratory and therefore such patients are excluded from this analysis.

If a patient had multiple isolates during the study period, those with identical susceptibility profiles were regarded as a single isolate. For patients with multiple isolates with varying susceptibility profiles, only the most resistant isolate was selected. If specimens from patients were received from more than one hospital, then the most resistant isolate was used for analysis and attributed to the hospital of first contact. Patients with missing drug susceptibility data and/or hospital location were excluded from this analysis. Numbers of MDR patients referred to KGV Hospital were compared with the number of patients that were diagnosed to ascertain referral and treatment rates. Data were not available on clinical symptoms, tuberculosis treatment outcome, new vs. re-treatment cases or HIV status.

### Definitions

MDR tuberculosis was defined as resistance to isoniazid and rifampin, with or without resistance to additional anti-tuberculosis drugs. XDR tuberculosis was defined as MDR tuberculosis with additional resistance to kanamycin and ofloxacin. The term MDR tuberculosis includes XDR tuberculosis cases, unless specified otherwise.

### Statistical analysis

MDR and XDR tuberculosis proportion was calculated as number of cases per 100 culture confirmed cases. The denominator to calculate the number of culture confirmed tuberculosis cases was obtained from the KZN laboratory database. Population data from the health statistics report of 2003 were used for the calculation of incidence [3]. No accurate population data were available for the study period. The incidence rates in two groups are compared using an incidence rate ratio and the p-value is calculated by assuming that under H_0_ the number of cases in group 1 follows a binomial distribution with *N* = total number of cases in both groups, and *p* = person-time in group1/total person-time in both groups.

After excluding the Umzinyati district, the remaining districts were separated into two groups which have distinctly different proportions of XDR cases. These groups were found by sequentially pooling the districts and using Fisher's Exact test to determine if the proportion of XDR cases were not significantly different across the districts within each group.

A generalized linear model (GLM) for the binomial family with a log-link was fitted to the data to estimate the risk ratios comparing the risk of XDR tuberculosis in different geographical regions.

The data analysis was conducted using R version 2.11.0, The R Foundation for Statistical Computing and STATA® 11.0, StataCorp College Station, Texas.

### Ethics Statement

The study was approved by the Biomedical Research Ethics Committee at the University of KwaZulu-Natal (#BCA274/09). As these samples were collected for routine clinical care, patients were not asked to give informed consent at the time of the clinical encounters. For the intentions of this study, there was no requirement for informed consent since all data used were previously collected during the course of routine medical care and did not pose any additional risks to the patients.

## Results

During the study period, *M.tuberculosis* was cultured from 20858 patients in KwaZulu-Natal Province. Of these, 4170 (20%) were infected with MDR tuberculosis isolates. Four hundred and forty three (11% of the MDR tuberculosis cases) displayed the XDR tuberculosis susceptibility profile. Overall, 1429 (34%) of the MDR tuberculosis cases were referred to KGV Hospital for further management. This included 212 (48%) of all XDR tuberculosis cases in the province during the study period.

The incidence of culture confirmed cases of tuberculosis in the province was 144.6 per 100.000 person-years. However, this varied widely between districts (Table 1). The highest incidence was found in eThekwini (236.6/100.000 person-yrs) and the lowest in Amajuba (20.4/100.000 person-yrs). The incidence of culture confirmed MDR and XDR tuberculosis in the province was 28.9 and 3.1/100.000 person-years respectively (Table 1). The highest incidence of both MDR (77.2/100.000 person-years) and XDR (34.1/100.000 person years) tuberculosis was found in Umzinyati district, which includes the Msinga sub-district. When the cases found in the Msinga sub-district were removed from those in the remainder of the district, the MDR incidence dropped to a level comparable with other districts. However, the XDR incidence remained at 5.0 versus 1.5/100.000 person years, significantly higher (Incidence rate ratio: 3.36, p-value<0.0001).

**Table 1:** Incidence of culture-confirmed cases of newly-diagnosed tuberculosis, MDR tuberculosis and XDR tuberculosis in the 11 health districts of KwaZulu-Natal province.

District	Population	Incidence of new TB(per 100.000 person-yrs)	Incidence of MDR(per 100.000 person-yrs)	Incidence of XDR(per 100.000 person-yrs)
**Amajuba**	477,472	20.4	6	0.3
**eThekwini**	3,152,405	236.6	33.7	1.9
**iLembe**	571,686	103.9	19.1	1
**Sisonke**	304,409	103.8	20.6	2
**uGu**	718,221	72.7	23.8	0.9
**uMgungundlovu**	946,545	210.8	32.3	4
**uMkhanyakude**	584,898	111.6	50.8	0.3
**Umzinyati**	465,660	213	77.2	34.1
**uThukela**	670,226	39.6	6.8	0.8
**uThungulu**	903,822	76.9	21.8	1
**Zululand**	820,661	38.7	17.3	0.3
**Total**	9,616,005	144.6	28.9	3.1

The proportion of MDR cases among culture confirmed patients with tuberculosis varied per district from 14.2% in eThekwini to 45.6% in uMkhanyakude. Umzinyati district that includes the Msinga sub-district, ranked third highest with 36.2% (Table 2).

**Table 2:** Proportion of MDR and XDR cases of tuberculosis in the 11 health districts of KwaZulu-Natal province (as a proportion of culture-confirmed TB cases).

District	Number of CultureConfirmed TB cases	Proportion of MDR(%) cases	Proportion of XDR(%) cases
**Amajuba**	146	29.5	1.4
**eThekwini**	11188	14.2	0.8
**iLembe**	891	18.4	1
**Sisonke**	474	19.8	1.9
**uGu**	783	32.7	1.3
**uMgungundlovu**	2993	15.3	1.9
**uMkhanyakude**	979	45.6	0.3
**Umzinyati**	1488	36.2	16
**uThukela**	398	17.1	2
**uThungulu**	1042	28.4	1.3
**Zululand**	476	44.7	0.8
**Total**	20858	20	2.1

The proportion of XDR tuberculosis cases in the Umzinyati district was 16%; this figure ranged from 0.3% to 2.0% in the other 10 districts (Table 2). The proportion of XDR tuberculosis in the Msinga sub-district was 19.6% vs 5.9% in the remaining part of the Umzinyati district. This last figure is approximately 3 times higher than in the district with the second highest proportion (Table 2).

When Umzinyati was excluded, the ten remaining districts formed two distinct groups based on the proportion of XDR cases (Table 3). Group 1 had an average proportion of XDR of 1.9% (range 1.9 to 2.0) while in group 2 the average was 0.8% (range 0.3 to 1.4). Compared with the Umzinyati district (without Msinga sub-district), this translated to a Risk Ratio of 0.33 (95% CI 0.21–0.51) and 0.14 (95% CI: 0.09–0.22) respectively (p<0.0001). All 3 districts in group 1 are adjacent to each other and located southwest of Umzinyati. The 7 districts of group 2 are in the north of the province and along the coast (Figure 1).

**Table 3:** Health districts according to proportion of culture-confirmed XDR tuberculosis.

District	Number of CultureConfirmed TB cases	Number of XDR cases	Proportion of XDR(%) cases
**Msinga sub-district**	1096	215	19.6
**Umzinyati – without Msinga sub-district**	392	23	5.9
**Group 1- Sisonke, uMgungundlovu, uThukela**	3865	74	1.9*
**Group 2- uMkhanyakude, eThekwini, Zululand, iLembe, uGu, uThungulu, Amajuba**	15505	131	0.8**

*Range: 1.9–2.0;

**Range: 0.3–1.4.

**Figure 2:**
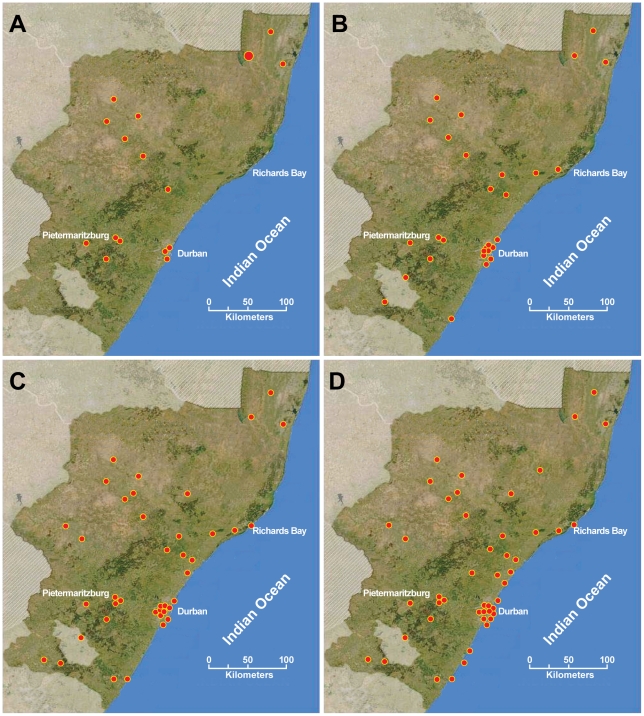
Geographical distribution and increase in number of hospitals with at least one case of XDR tuberculosis between January 2006 and June 2007. Each red dot depicts one hospital.

On the 1^st^ of January 2006, patients infected with XDR tuberculosis had been detected in 16 (22%) hospitals, which increased to 58 (78%) by the end of June 2007. The geographical distribution of this increasing number of hospitals over time is shown in figure 2.

## Discussion

We report on the temporal spread of XDR tuberculosis in KwaZulu-Natal, the province of South Africa from which the first report on XDR tuberculosis emerged [2]. The data show that in January 2006, XDR patients were detected in 16 hospitals located in five provincial districts. By the end of the 18-month study period, this figure extended to 58 hospitals involving all 11 districts.

The reported incidence of tuberculosis in 2006 in KZN was 780/100,000 [1]. We found the incidence of culture-confirmed disease to be 145/100,000. This highlights the fact that over 80% of reported cases of tuberculosis are not culture-confirmed and, by default, also lack drug susceptibility test results. Although this may be in keeping with the policy on diagnosis of tuberculosis in South Africa, it is a matter of concern since a significant proportion of patients with MDR/XDR tuberculosis are likely to die before it is realized that drug susceptibility tests are needed to guide their treatment. An additional concern is that more than 65% of MDR (including XDR) cases of culture-confirmed tuberculosis did not reach the referral centre and therefore remained untreated. These large groups of undiagnosed and diagnosed-but-not-referred cases of drug-resistant tuberculosis form a contingent of patients whose disease is inappropriately managed. They also represent a pool of potential transmitters of drug-resistant tuberculosis in the community or in hospitals.

The last drug resistance survey in South Africa in 2001/2002 found that the MDR tuberculosis prevalence in KwaZulu-Natal was 1.7% among new patients and 7.7% among retreatment patients. This was similar to other provinces [6]. Second-line drug susceptibility testing was not performed in this survey and therefore the proportion of XDR cases is unknown. However, XDR was already present in KwaZulu-Natal Province in 2001 [7]. We show an overall increase in the proportion of culture-confirmed MDR to 20% (Table 1). However, this figure cannot be compared with those found during the 2001/02 survey. The reported prevalence in 2001/02 is based on active case finding in randomly chosen areas in the province and reflects the situation amongst all patients with tuberculosis. Our figures represent only the proportion amongst culture-confirmed cases in the province and are therefore a gross underestimation of the total number of MDR cases in KZN. Despite this underestimation, we show a doubling of MDR cases over the 5-year period from 2001/02 to 2006.

The proportion of culture-confirmed XDR tuberculosis in the Umzinyati district was more than seven times higher than that in the rest of the province. Although this was largely attributable to the high prevalence in the Msinga sub-district where all suspected cases are cultured, the observation that the remaining part of the Umzinyati district with culture practices comparable to the other districts also had a higher proportion of XDR cases than the rest of the province (Table 3) alludes to circumstances in that district that may have facilitated the emergence of XDR tuberculosis.

The data also show that the presence of XDR tuberculosis is not restricted to the Umzinyati district. Although the proportion of XDR amongst culture-confirmed cases is lower in the other districts (Table 2), this might not reflect the real situation. Patients with tuberculosis from the Msinga sub-district obtained a diagnosis through culture and drug susceptibility testing on first presentation. As a result, a diagnosis of drug-resistant tuberculosis was already made even if patients died in the ensuing weeks. In the rest of the province, cultures were done when there was no response to treatment. As many patients with XDR tuberculosis die within weeks from first presentation [2], a significant number of patients in other parts of the province may have remained undiagnosed.

Whole genome sequencing data on a limited number of randomly chosen XDR isolates from KwaZulu-Natal Province suggest that one XDR strain has spread throughout the province [8]. This needs confirmation by sequencing of a larger number of isolates. The observation that the proportion of XDR cases in the Umzinyati district (even without Msinga) is higher than that in the other districts suggests that this may be the source of such clonal spread. This is supported by the observation that the three districts in the southwest of the province had a higher proportion of XDR patients as compared to the other districts. Like the communities of Umzinyati, people from these districts use Pietermaritzburg as their main shopping and cultural centre. The other districts use Durban and Empangeni for that purpose. The observed temporal increase of XDR throughout the province during the study period also supports the possibility of clonal spread. However, this is a retrospective study and no concrete conclusions can be made regarding the true nature of the acquisition and spread of TB in this group of patients.

Since there is not, as yet, effective treatment for XDR tuberculosis, tuberculosis control programmes need to include more effective strategies to curb its spread. A rapid and efficient implementation of the new national guidelines for the initiation of anti-retroviral treatment at a CD4 count of 350/mm^3^ as opposed to 200/mm^3^ would assist in decreasing the size of the susceptible population [9]. In addition, the infection prevention strategies to limit transmission in health care facilities as well as in the community need to be implemented and evaluated. Without this, the implementation of the new policy of combined care for patients with active tuberculosis and HIV infected patients without tuberculosis is likely to promote further spread of tuberculosis. The development of a rapid diagnostic test that is able to detect the presence of *M. tuberculosis* and its susceptibility to first-line and second-line drugs within a couple of hours should have the highest priority. Such a test will be able to identify MDR and XDR cases much earlier and facilitate more efficient management of this disease and earlier implementation of strategies to prevent transmission of drug-resistant tuberculosis.
